# Performance of COPD population screener questionnaire in COPD screening: a validation study and meta-analysis

**DOI:** 10.1080/07853890.2021.1949486

**Published:** 2021-07-20

**Authors:** Yanhui Gu, Ying Zhang, Qian Wen, Yao Ouyang, Yongchun Shen, He Yu, Chun Wan, Jing Zhu, Fuqiang Wen

**Affiliations:** a Department of Respiratory and Critical Care Medicine, West China Hospital of Sichuan University, Chengdu, China; b Department of Respiratory and Critical Care Medicine, Affiliated Hospital of Zunyi Medical University, Zunyi, China; c Health Management Center, Affiliated Hospital of Zunyi Medical University, Zunyi, China

**Keywords:** Chronic obstructive pulmonary disease, chronic obstructive pulmonary disease population screener, screening, meta-analysis

## Abstract

This study aimed to validate the chronic obstructive pulmonary disease (COPD) Population Screener (COPD-PS) questionnaire as a screening tool in a cohort of Chinese subjects who underwent a health examination, and to summarise its overall performance through a meta-analysis. We enrolled 997 subjects aged ≥40 years who underwent a health examination, both lung function and COPD-PS data were collected. The screening performance of COPD-PS was evaluated with a receiver operating characteristic (ROC) curve analysis, using the area under the curve (AUC) to assess the screening accuracy. A standard diagnostic meta-analysis was used to summarise the screening performance of COPD-PS for COPD. Of the 997 subjects, 157 were identified as having COPD. The COPD-PS score was significantly higher in COPD patients than controls (5.03 ± 5.11 vs. 2.72 ± 1.80, *p* < .001). At a cut-off of 4, the sensitivity and specificity of COPD-PS for identifying COPD were 74.52 and 70.24%, respectively, with an AUC of 0.79. Eight studies (including this study) were included in this meta-analysis. The pooled estimates for COPD-PS were as follows: sensitivity of 0.66 (95% CI: 0.47–0.63), specificity of 0.86 (95% CI: 0.84–0.89), positive likelihood ratio of 3.00 (95% CI: 1.65–5.47), negative likelihood ratio of 0.43 (95% CI: 0.35–0.52) and diagnostic odds ratio of 7.24 (95% CI: 3.91–13.40). The AUC of the summary ROC curve was 0.78. COPD-PS appears to be a useful tool for screening individuals with a high risk of COPD and guiding the selection of individuals for subsequent spirometry examination.

**KEY MESSAGES**
- COPD-PS is a simple and useful method to screen COPD.
- The combination of COPD-PS with other tools may improve the screen performance.

## 1. Introduction

Chronic obstructive pulmonary disease (COPD) is a common disease that involves persistent respiratory symptoms and irreversible airflow limitation, and it is raising great public concern worldwide [1,2]. Data from a cross-sectional study in China revealed that the overall prevalence of spirometry-defined COPD was 8.6%, corresponding to 99.9 million people with COPD in China [3]. COPD is one of the most important causes of mortality worldwide, and it poses heavy economic and social burdens. In 2003, COPD was the third leading cause of mortality in the USA [4]. The direct and indirect costs of COPD were about 32 billion and 20.4 billion US dollars per year, respectively [4]. Considering the irreversible process of COPD, early identification of subjects at high risk of COPD and early diagnosis of COPD may help to improve the comprehensive management of patients with COPD, reduce symptoms, improve health status, prevent acute exacerbations, and ultimately, reduce mortality.

A standard lung function test using spirometry is the “gold standard” method to diagnose COPD [4]. However, there are several challenges to lung function test in China. First, there is a lack of equipment for spirometry in China, with the equipment mainly being located in tertiary hospitals rather than in hospitals of all levels, and the quality of spirometry tests in tertiary hospitals requires improvement [5,6]. Second, there is no evidence that conducting spirometry tests to diagnose COPD in asymptomatic individuals improves health-related quality of life, morbidity, or mortality, so spirometry tests for early detection in the general population without preselection of at-risk patients may waste healthcare resources [7,8]. A simple questionnaire or tool may help to identify cases with a high risk of airflow limitation, enhance the detection rate of COPD, and optimise the allocation of medical resources.

In 2008, a Clinician Working Group in the US developed a simple and self-administered questionnaire, the COPD Population Screener (COPD-PS), to screen for subjects in the general population at high risk of COPD. It consists of an item on age, an item on cigarette smoking, and three COPD-related items (breathlessness, productive cough and activity limitation) [9]. In recent years, COPD-PS has been validated in several countries and it appears to be an adequate tool for large-scale screening for COPD requiring further spirometry testing [10,11]. As there is no report on the utility of COPD-PS in Chinese, this study aimed to investigate the screening performance of COPD-PS in a Chinese cohort undergoing a health examination, and to summarise the overall screening accuracy of COPD-PS for identifying COPD cases using a meta-analysis.

## 2. Patients and methods

### 2.1. Patients

Subjects who underwent a health examination at the Health Management Centre of the Affiliated Hospital of Zunyi Medical University (Zunyi, China) were prospectively recruited between June and September 2020. Subjects aged ≥40 years without known chronic respiratory diseases (including bronchiectasis, asthma and COPD) were enrolled. Subjects with acute respiratory symptoms or malignancies such as lung cancer were excluded. All the subjects signed an informed consent form. The study was approved by the Ethics Committee of the Affiliated Hospital of Zunyi Medical University (no. 2018-45) and was conducted based on the principles outlined in the Declaration of Helsinki.

### 2.2. Data collection

The subjects completed a simple questionnaire to collect the following demographic and clinical data: age, sex, cigarette smoking history, body mass index (BMI, kg/m^2^), lung function with forced expiratory volume in the first second (FEV_1_), forced vital capacity (FVC), FEV_1_/FVC ratio and FEV_1_% Pred.

### 2.3. Spirometry examination

First, the subjects underwent a simple spirometry examination (Medikro Pro spirometer, Medikro Oy, Finland) focussed on lung ventilation function conducted by well-trained technicians. Then, subjects with pre-bronchodilator FEV_1_/FVC <0.7 underwent a standard post-bronchodilator spirometry examination (MasterScreen, CareFusion, Germany). COPD was defined as post-bronchodilator FEV_1_/FVC <70%, according to the Global Initiative for Chronic Obstructive Lung Disease (GOLD) criteria [4].

### 2.4. COPD-PS questionnaire

The subjects completed the Chinese version of the COPD-PS questionnaire by themselves. If they had any questions on the questionnaire, a trained physician helped them to complete it. The COPD-PS questionnaire consists of five items, three on COPD-related symptoms (5-point scale), one on cigarette smoking (3-point scale) and one on the subject’s age (four categories). These five items are then scored 0, 1 or 2 with a summed total score ranging from 0 to 10 [9]. Calculation of the COPD-PS scores was performed by two independent blinded investigators using predetermined scoring criteria.

### 2.5. Statistical analysis

The data are presented as mean ± standard deviation. Intergroup differences were assessed for significance using the Mann–Whitney *U* test. A receiver operating characteristic (ROC) curve and the area under the ROC curve (AUC) were used to assess the screening performance of COPD-PS for COPD. Further, we determined the COPD-PS score cut-off associated with the sensitivity and specificity optimising Youden’s index [12]. The statistical analyses were performed in SPSS 21.0 (IBM Corp., Armonk, NY), and differences with *p* < .05 were considered statistically significant.

### 2.6. Meta-analysis

The meta-analysis was performed according to the Preferred Reporting Items for Systematic Reviews and Meta-Analyses (PRISMA) statement and methods recommended by the Cochrane Diagnostic Test Accuracy Working Group [13]. We used PubMed, Scopus and Web of Science to systematically identify studies on COPD-PS published before July 2020. The following search terms were used in each database: “Chronic obstructive pulmonary disease”, “COPD”, “COPD-PS”, “COPD Population Screener,”, “sensitivity”, “specificity” and “accuracy”. We included original clinical research articles that reported true positive (TP), false positive (FP), false negative (FN) and true negative (TN) data on the use of COPD-PS for identifying COPD. Only articles published in English were considered. Two reviewers (YG and YZ) independently evaluated the studies initially based on the titles and abstracts and subsequently based on the full text. They assessed the quality of the included studies using a revised version of QUADAS-2 [14].

We calculated pooled estimates of sensitivity, specificity, positive likelihood ratio (PLR), negative likelihood ratio (NLR) and diagnostic odds ratio (DOR), along with 95% confidence intervals (CIs). Heterogeneity among the included studies was evaluated based on Cochran’s *Q* test and *I*^2^ examination, *I*^2^ > 50% indicated significant heterogeneity and a random effects model was used to pool the data, and *I*^2^ < 50% indicated low risk of heterogeneity then a fixed effects model was used to pool the data [15,16]. The overall screening performance of COPD-PS was assessed based on the summary ROC (SROC) curve. Potential publication bias was evaluated using Deeks’ test [17]. All meta-analysis procedures were performed by using Stata 15.0 (Stata Corp., College Station, TX) and Meta-DiSc 1.4 for Windows (XI, Cochrane Colloquium, Barcelona, Spain) [18,19]. *p* < .05 indicated statistical significance.

## 3. Results

### 3.1. Characteristics of the included subjects

There were 997 subjects included in this study, with 157 cases of COPD, corresponding to an incidence of 15.74%. The mean age of the subjects was 59.12 ± 11.75 years, and the percentage of males was 56.57% (564/997). In the COPD group, the patients had a higher age (64.75 ± 10.48 vs. 58.06 ± 11.68, *p* < .001), a higher percentage of males (71.34 vs. 53.81%, *p* < .001) and a higher percentage of current/ever smokers (67.52 vs. 47.61%, *p* < .001) compared to the control group. The numbers of COPD patients with GOLD stages I, II, III and IV were 45, 75, 27 and 10, respectively. The clinical characteristics of included subjects are summarised in Table 1.

**Table 1.** Clinical characteristics of included subjects.

	COPD	Control	*p* Value
Number	157	840	
Age （year）	64.75 ± 10.48	58.06 ± 11.68	<.001
Gender			
Male	112	452	<.001
Female	45	388	
BMI	22.36 ± 3.55	23.58 ± 3.55	<.001
Lung function test			
FEV1 (L)	1.56 ± 0.67	2.45 ± 0.71	<.001
FVC (L)	2.63 ± 0.89	3.09 ± 0.86	<.001
FEV1/FVC (%)	57.71 ± 8.86	79.56 ± 5.46	<.001
FEV1 Pred%	66.76 ± 22.12	99.51 ± 17.26	<.001
Smoking status			
Never smoker	51	440	<.001
Ever smoker	39	113	
Current smoker	67	288	
GOLD stage			
I	45	—	
II	75	—	
III	27	—	
IV	10	—	
COPD-PS score	5.03 ± 2.11	2.72 ± 1.80	<.001

COPD-PS: chronic obstructive pulmonary disease population screener; FEV_1_: forced expiratory volume in the first second; FVC: forced vital capacity; GOLD: global initiative for chronic obstructive lung disease.

### 3.2. Screening performance of COPD-PS

**Figure 1.**
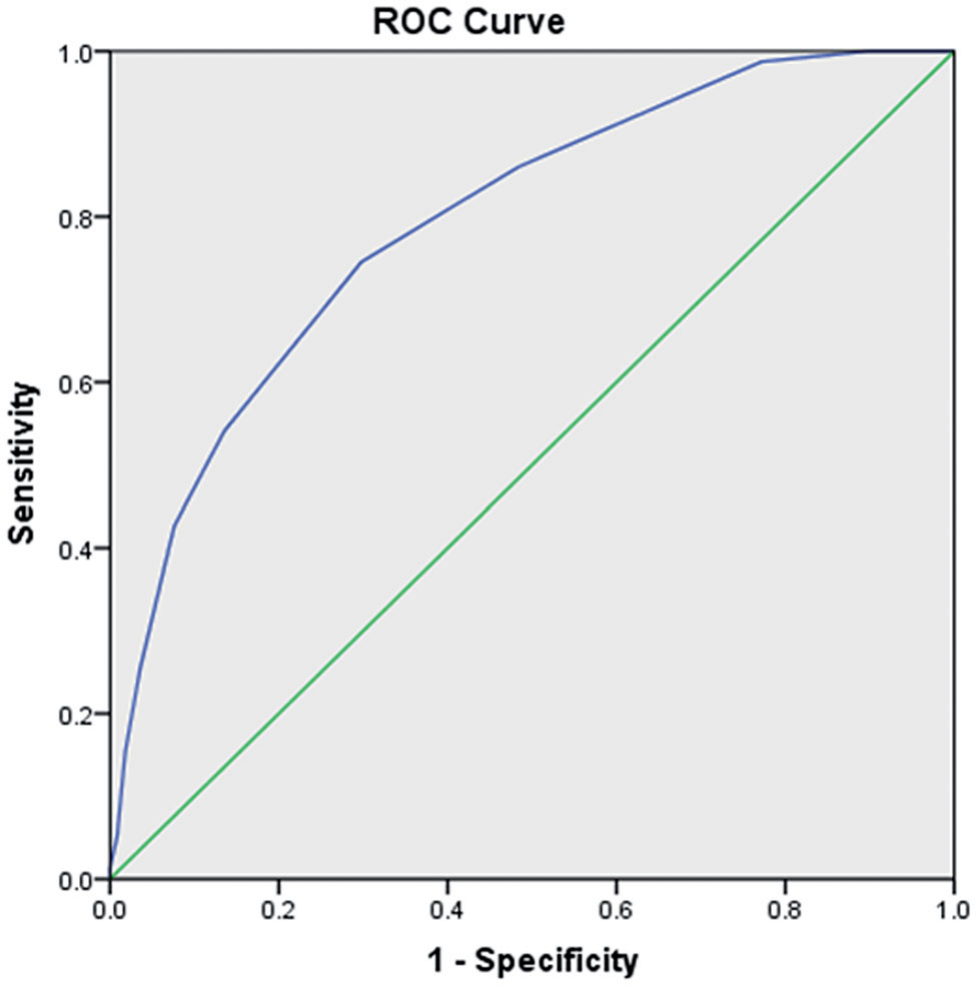
Receiver operating characteristic (ROC) curve of the use of COPD-PS to discriminate between COPD patients and controls. COPD-PS: chronic obstructive pulmonary disease population screener.

The COPD-PS score was significantly higher in patients with COPD than in subjects without COPD (5.03 ± 2.11 vs. 2.72 ± 1.80, *p* < .001). The COPD-PS score increased with the GOLD stage (*p* < .001). A ROC curve was created to summarise the screening performance of COPD-PS for COPD, and the AUC was 0.79 (Figure 1). As the cut-off was raised, the sensitivity of COPD-PS decreased, while the specificity increased (Table 2). At the optimum cut-off of four points, COPD-PS had a sensitivity of 74.52% and a specificity of 70.24%.

**Table 2.** Performance of COPD-PS in detecting COPD with various cut-off values.

Cut-off value	Sensitivity (%)	Specificity (%)	Positive predictive value (%)	Negative predictive value (%)
3	85.99	51.67	24.95	95.18
4	74.52	70.24	31.88	93.65
5	54.78	86.43	43.00	91.09
6	43.31	92.38	51.52	89.71
7	26.11	96.43	57.75	87.47

### 3.3. Meta-analysis

**Figure 2.**
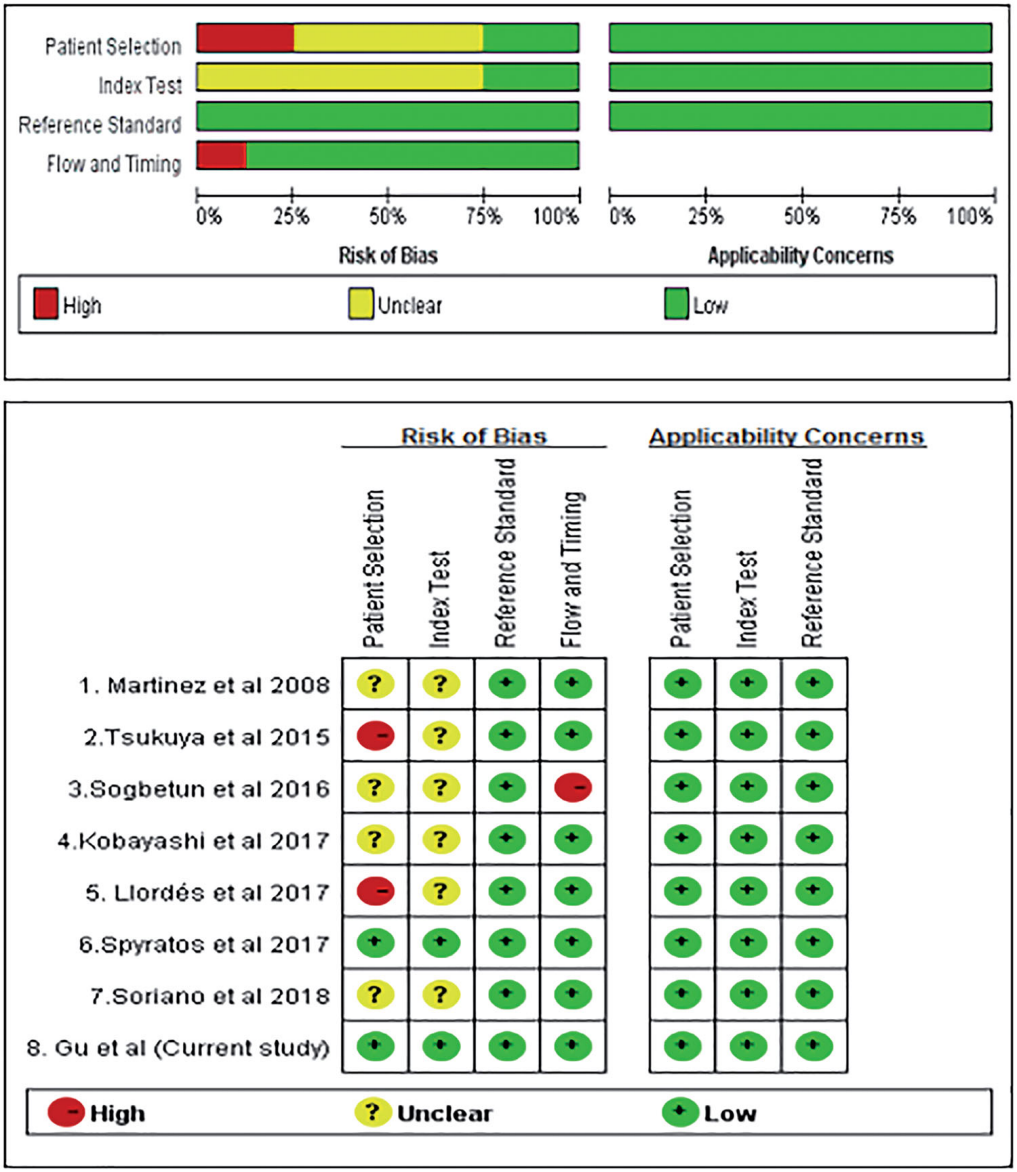
Quality assessment of studies on COPD-PS. COPD-PS: chronic obstructive pulmonary disease population screener.

Next, we performed a meta-analysis of a total of eight studies (including this study) involving 1139 patients with COPD and 13,555 controls [9,10,20–24]. These studies were published from 2008 to 2018, across five countries (two in the USA, two in Japan, two in Spain, one in Greece and one in China). The subjects were from primary care centres/hospitals (*n* = 4), the general population (*n* = 3) and a health examination centre (*n* = 1). All the subjects underwent spirometry examination. The clinical characteristics of the patients are listed in Table 3. The quality assessment of the included studies is shown in Figure 2.

**Table 3.** Clinical summary of included studies.

Author	Study number	Year	Country	COPD	GOLD (I/II/III/IV)	Control	Source of subjects	Age requirement	COPD definition	Cut-off	TP	FP	FN	TN
Martinez et al. [9]	1	2008	USA	113	NA	182	GP/SS	≥35	Spirometry	6	83	48	30	134
Tsukuya et al. [10]	2	2015	Japan	153	71/73/8/1	2204	General population	40–79	Spirometry	4	103	597	50	1607
Sogbetun et al. [20]	3	2016	USA	101	NA	261	Primary care clinics	NA	Spirometry	5	74	144	27	117
Kobayashi et al. [21]	4	2017	Japan	27	NA	46	Primary care clinics and hospitals	≥40	Spirometry	5	17	15	10	31
Llordés et al. [22]	5	2017	Spain	107	NA	300	General population	≥45	Spirometry	4	86	157	21	143
Spyratos et al. [23]	6	2017	Greece	351	NA	2883	General population	>40	Spirometry	5	196	288	155	2595
Soriano et al. [24]	7	2018	Spain	130	NA	6839	Primary care centres	≥35	Spirometry	4	108	546	22	6293
Gu et al. (current study)	8	—	China	157	45/75/27/10	840	Subjects underwent health examination	≥40	Spirometry	4	117	250	40	590

COPD: chronic obstructive pulmonary disease; FN: false negative; FP: false positive; GOLD: Global Initiative for Chronic Obstructive Lung Disease; TN: true negative; TP: true positive; NA: not available.

**Figure 3.**
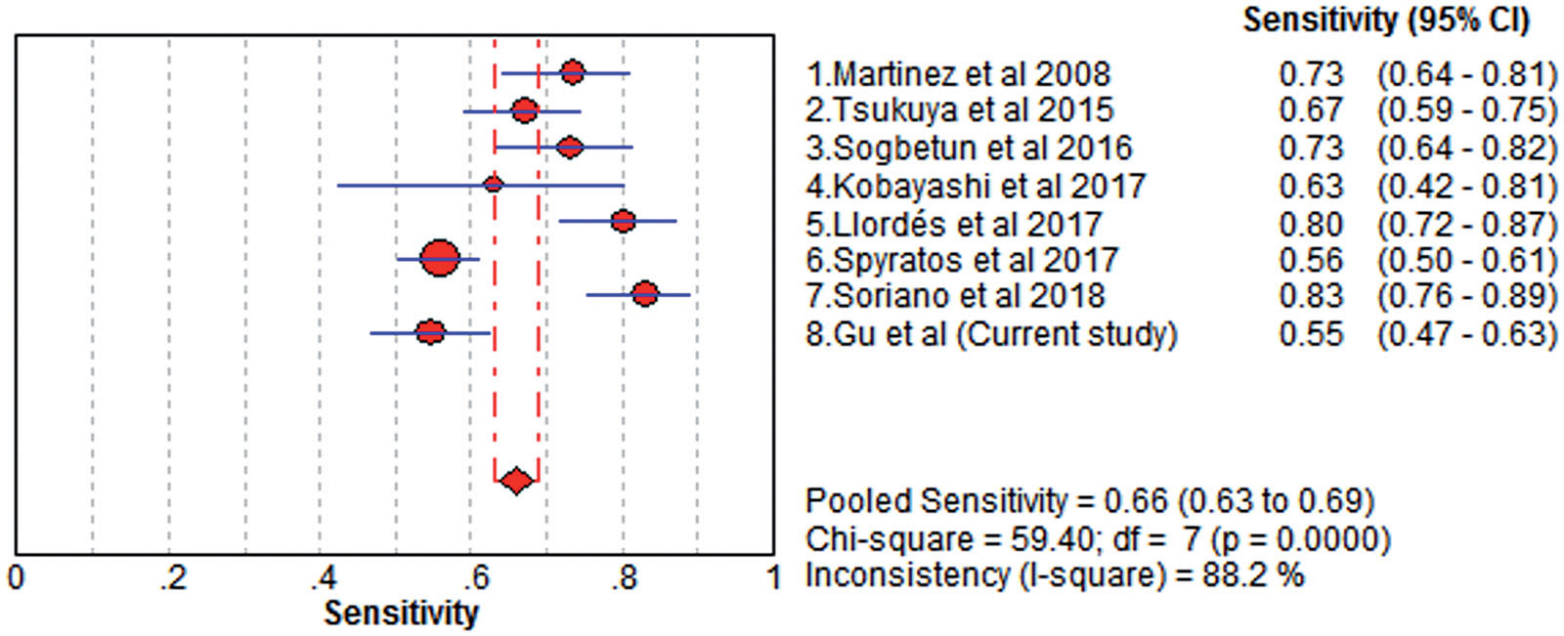
Forest plot of sensitivity COPD-PS with random-effects model. The point estimates of sensitivity from each study are shown as solid circles. Error bars indicate 95% CIs. COPD-PS: chronic obstructive pulmonary disease population screener.

**Figure 4.**
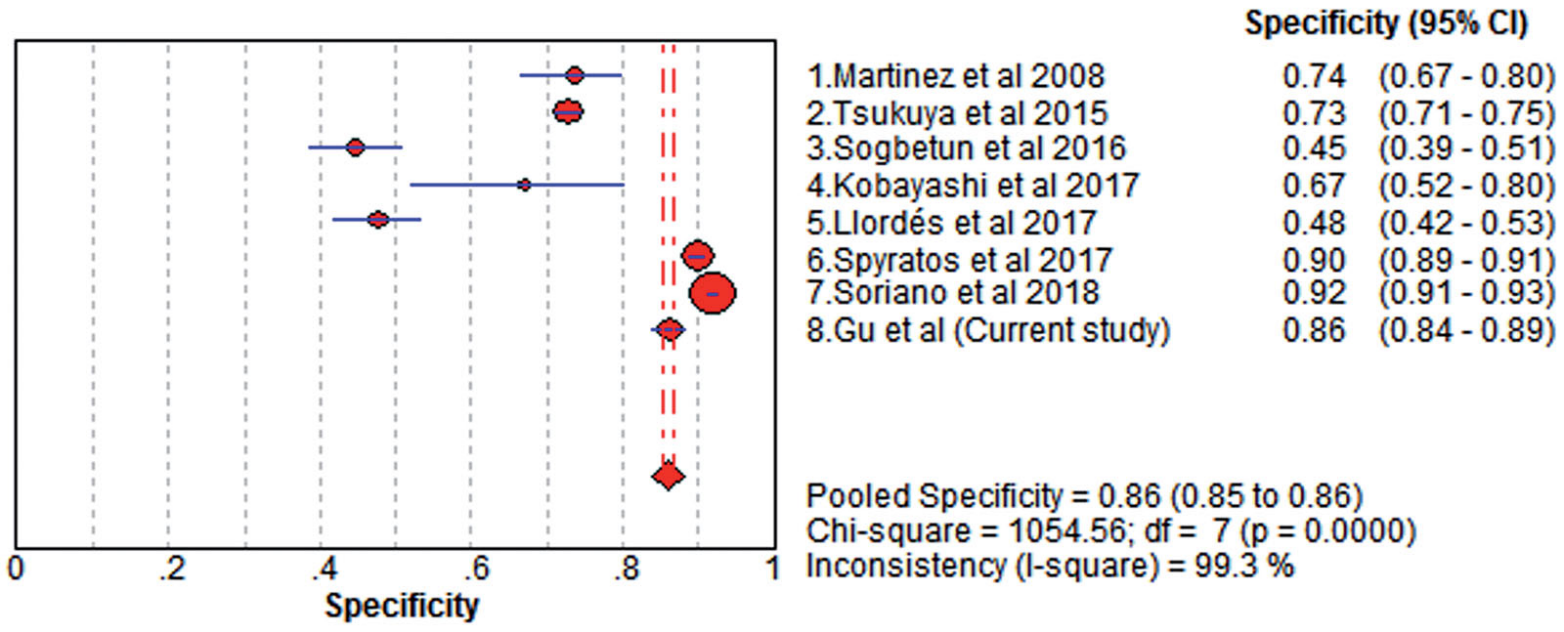
Forest plot of specificity of COPD-PS with random-effects model. The point estimates of specificity from each study are shown as solid circles. Error bars indicate 95% CIs. COPD-PS: chronic obstructive pulmonary disease population screener.

**Figure 5.**
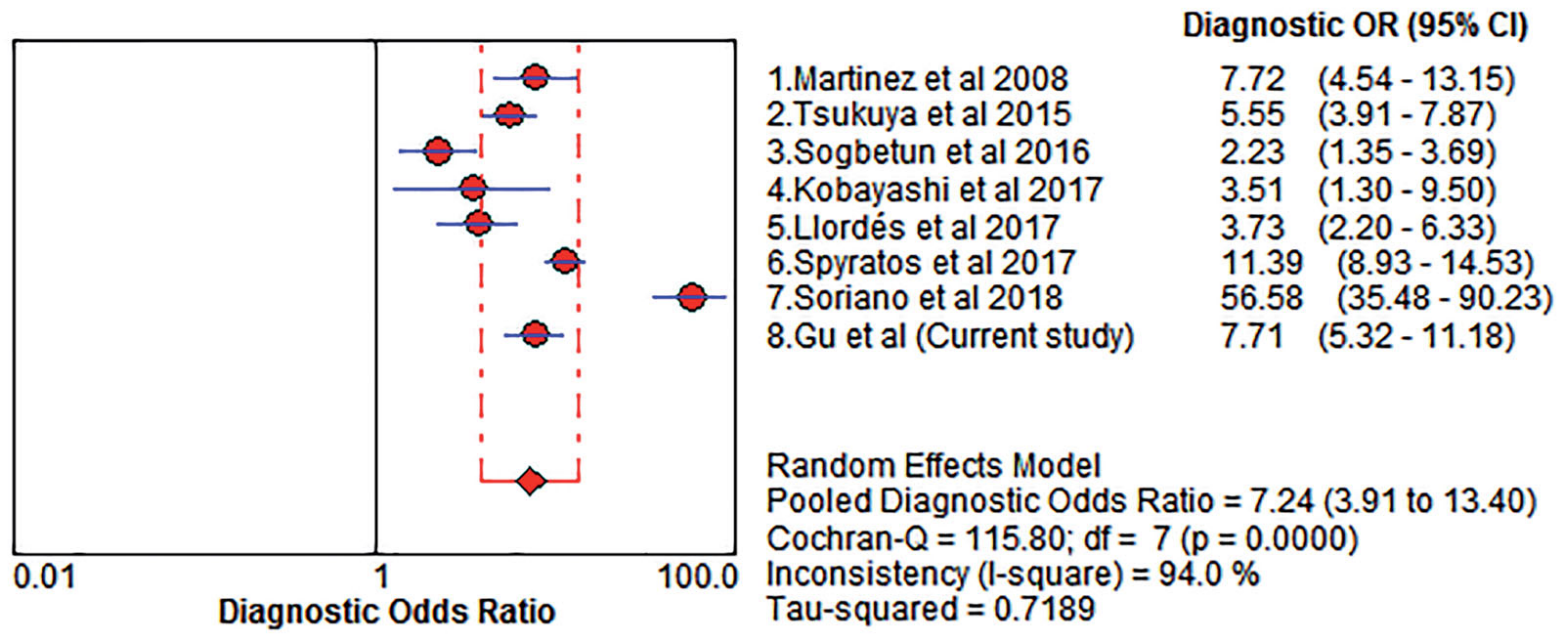
Forest plot of diagnostic odds ratio of COPD-PS with random effects model. The point estimates of specificity from each study are shown as solid circles. Error bars indicate 95% CIs. COPD-PS: chronic obstructive pulmonary disease population screener.

**Figure 6.**
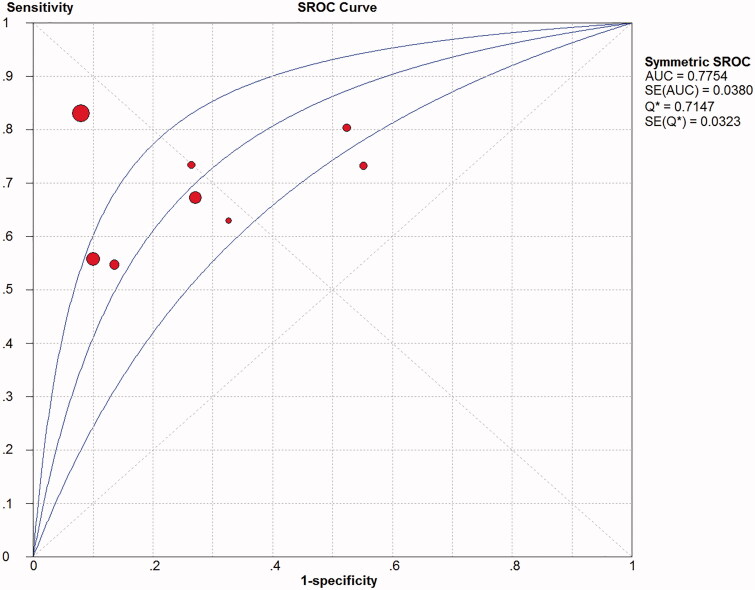
Summary receiver operating characteristic (SROC) curve of COPD-PS as a screening tool for COPD. COPD-PS: chronic obstructive pulmonary disease population screener. Q: The maximum joint value of sensitivity and specificity of COPD-PS.

There was significant heterogeneity among included studies with respect to the sensitivity (*I*^2^ = 88.20%; *p* < .05) and specificity (*I*^2^ = 99.30%; *p* < .05). Thus, the random effects model was chosen to pool the data. The accuracy of COPD-PS for identifying COPD was assessed, with pooled estimates of sensitivity of 0.66 (95% CI: 0.47–0.63) (Figure 3), specificity of 0.86 (95% CI: 0.84–0.89) (Figure 4), PLR of 3.00 (95% CI: 1.65–5.47), NLR of 0.43 (95% CI: 0.35–0.52) and DOR of 7.24 (95% CI: 3.91–13.40) (Figure 5). The SROC curve showed that the AUC was 0.78 and the *Q* value was 0.71 (Figure 6). The results of fixed effect model were also presented in Supplementary Material 1.

**Figure 7.**
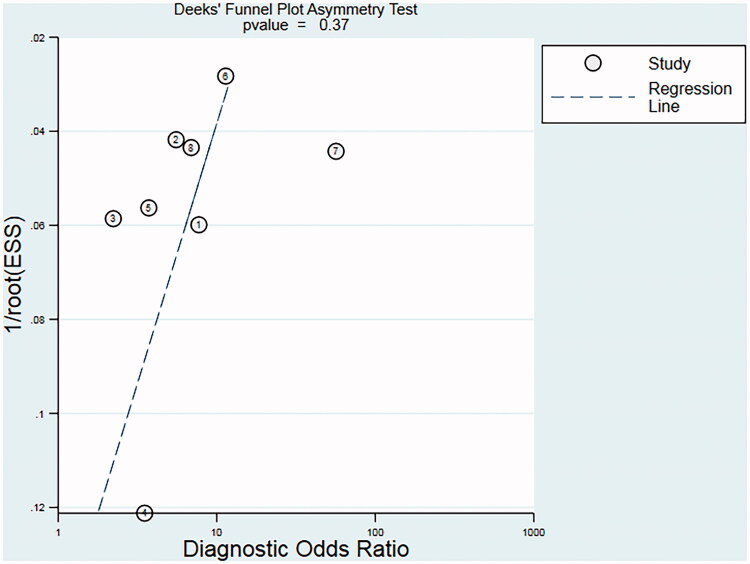
Deeks’ funnel plot assessing the likelihood of publication bias. The statistically nonsignificant *p*-value of 0.37 for the slope coefficient suggests symmetry in the data and a low likelihood of publication bias. ESS: effective sample size, corresponding to 4 × *N*control × *N*_COPD_/(*N*control + *N*_COPD_).

Although significant heterogeneity among included studies was identified, a meta-regression analysis was not performed to investigate the source of heterogeneity due to the limited number of included studies. There was low likelihood of publication bias based on Deeks’ funnel plot (*p* = 0.37; Figure 7).

## 4. Discussion

Identifying a simple method to screen COPD is of great importance to find COPD cases early and optimise medical resource allocation, and thus improve the management of patients. In this study, we validated COPD-PS in a Chinese cohort of health examination, and we also found that COPD-PS could play a role in screening for COPD. These findings were further confirmed by our meta-analysis.

The COPD-PS score was developed more than 10 years ago and has not previously been validated in a Chinese cohort. We performed a clinical study at a health examination centre in West China, and the results showed that the COPD-PS score is increased in patients with COPD. ROC analysis suggested that COPD-PS has the potential to identify COPD cases (AUC = 0.79). With increasing cut-off values, the sensitivity of COPD-PS score in screening for COPD decreased. At a cut-off of 4, the sensitivity and specificity of COPD-PS were 74.52 and 70.24%, respectively. The positive predictive value is 31.88%, which means that the probability that subjects with a positive COPD-PS screening test truly have COPD is quite low. The negative predictive value is 93.65%, which means that among those who had a negative COPD-PS screening test, the probability of being non-COPD was as high as 93.65%. When compared with other studies, the source of our subjects was from health examination centre at a tertiary care centre, not at primary care, and the incidence of COPD was as high as 15.74%. Thus, whether our results are acceptable for general population remains unclear. Additionally, in this study, we removed subjects with acute respiratory symptoms or malignant diseases, and how to screen COPD or whether COPD-PS is useful for such a population to remain unclear. Anyway, our results suggest that COPD-PS is a useful tool for identifying subjects at high risk of COPD.

The results of our meta-analysis of the screening performance of COPD-PS also showed moderate pooled estimates of sensitivity (0.66) and high specificity (0.86). The SROC curve, which assesses overall screening performance and depicts the trade-off between sensitivity and specificity, indicated an AUC of 0.78; *Q* value, the intersection point of the SROC curve with a diagonal line from the left upper corner to the right lower corner of the ROC space and corresponding to the maximum joint value of sensitivity and specificity for COPD-PS, is 0.71, both AUC and *Q* value suggest a moderate accuracy. The DOR value combines sensitivity and specificity data into a single number ranging from 0 to infinity; higher DOR values indicate better discriminatory performance. The mean DOR in our meta-analysis was 7.24, suggesting that COPD-PS is a useful COPD screening tool. Similarly, the pooled PLR value (3.00) suggested that COPD patients are approximately three times more likely to have a positive COPD-PS result than controls. The pooled NLR value (0.43) suggested that a subject has a 43% likelihood of having COPD even with a negative COPD-PS assessment. Importantly, COPS-PS is not a tool for making a final diagnosis of COPD, it is a screening tool to identify subjects at high risk of COPD who may need further spirometry examination. Based on the results of the meta-analysis, COPD-PS may be a useful tool for large-scale screening for COPD in the general population.

COPD-PS has several advantages. First, it is a simple and easy to use tool; there was no significant difference in the mean COPD-PS score between internet-based or paper and pencil-based methods, and the predictive utility of COPD-PS did not differ between methods of administration, even after accounting for age and smoking status [25]. Second, COPD-PS can be combined with other tools to improve the identification of COPD cases. For example, Soriano et al. reported that the combination of COPD-PS and peak expiratory flow (PEF) measurements at recommended thresholds in primary care is a useful, reliable strategy for finding new COPD cases, leading to a 90% reduction in the number of spirometry tests performed [24]. COPD-PS combined with COPD-6 could potentially be used by clinicians to identify individuals at risk of COPD and to select patients for spirometry measurement [26]. Thus, we suggest that COPD-PS should be used with other tools, which may substantially reduce the number of unnecessary spirometry tests. In addition, it is important to note that COPD-PS is a tool to screen for COPD, rather than to diagnose COPD; it cannot replace spirometry to make a final diagnosis of COPD.

This study has several limitations that should be noted. First, we enrolled subjects who underwent a health examination at a health examination centre in a tertiary care centre, in the meta-analysis, two of the studies only included smokers [22,23], these subjects do not represent the general population, so the results of this study should be interpreted with caution when applied to general population, or subjects at primary care, or subjects with specific smoke history. Second, we only included eight studies in this meta-analysis, which may not be enough to draw a final conclusion. Third, although the results of Deeks’ test suggested a low possibility of publication bias, it cannot conclude that there is no funnel asymmetry since only eight studies were included this meta-analysis [27]. Last but not least, we identified significant heterogeneity among included studies, the performance of the COPD-PS may be overestimated and should be treated with caution. So, current findings should be validated in more countries, location types (i.e. hospitals, etc.) and target groups with specific conditions to fully assess the role of COPD-PS as a screening tool.

In summary, COPD-PS is a simple tool that may be useful for screening for COPD. The combination of COPD-PS and other tools may help to improve screening performance for COPD.

## Supplementary Material

Supplemental Material

## Data Availability

All data used to support the findings of this study are available from the corresponding authors upon reasonable request.
