# Supplementary material for: Performance of COPD population screener questionnaire in COPD screening: a validation study and meta-analysis
Source: Ann Med. 2021 Jul 20;53(1):1199–207. doi: 10.1080/07853890.2021.1949486 (PMC8293944; doi:10.1080/07853890.2021.1949486)
Supplement: Supplemental Material [file IANN_A_1949486_SM0836.docx]

**Supplementary Figure 1. Forest plot of positive likelihood ratio of COPD-PS with fixed effects model.**


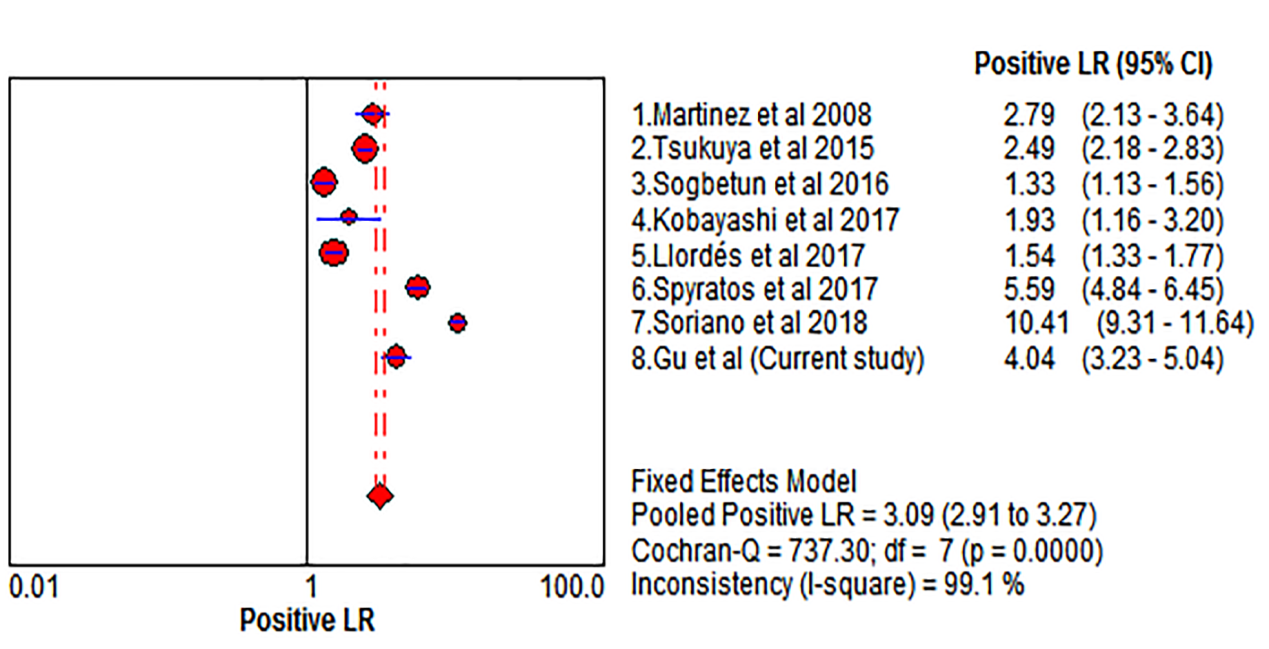


The point estimates of specificity from each study are shown as solid circles. Error bars indicate 95% CIs. I^2^ = (Q-df)/Q.

COPD-PS: Chronic Obstructive Pulmonary Disease Population Screener

**Supplementary Figure 2. Forest plot of negative likelihood ratio of COPD-PS with fixed effects model.**


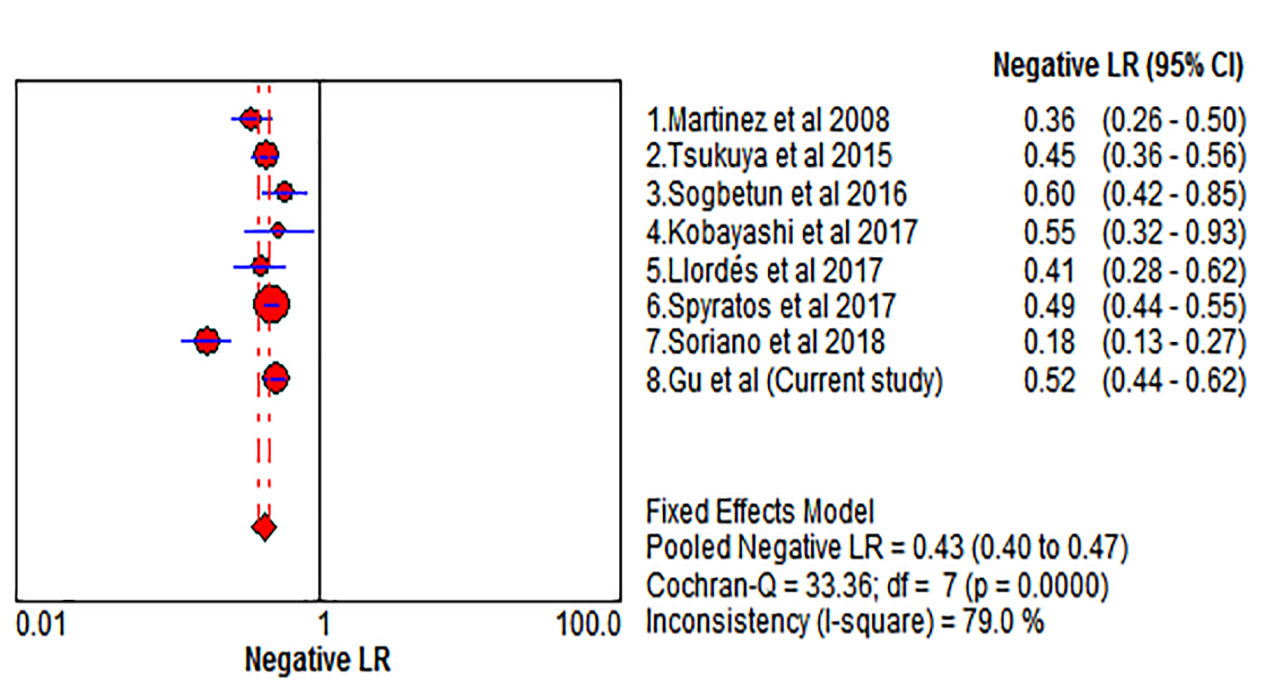


The point estimates of specificity from each study are shown as solid circles. Error bars indicate 95% CIs. I^2^ = (Q-df)/Q.

COPD-PS: Chronic Obstructive Pulmonary Disease Population Screener

**Supplementary Figure 3. Forest plot of diagnostic odds ratio of COPD-PS with fixed effects model.**


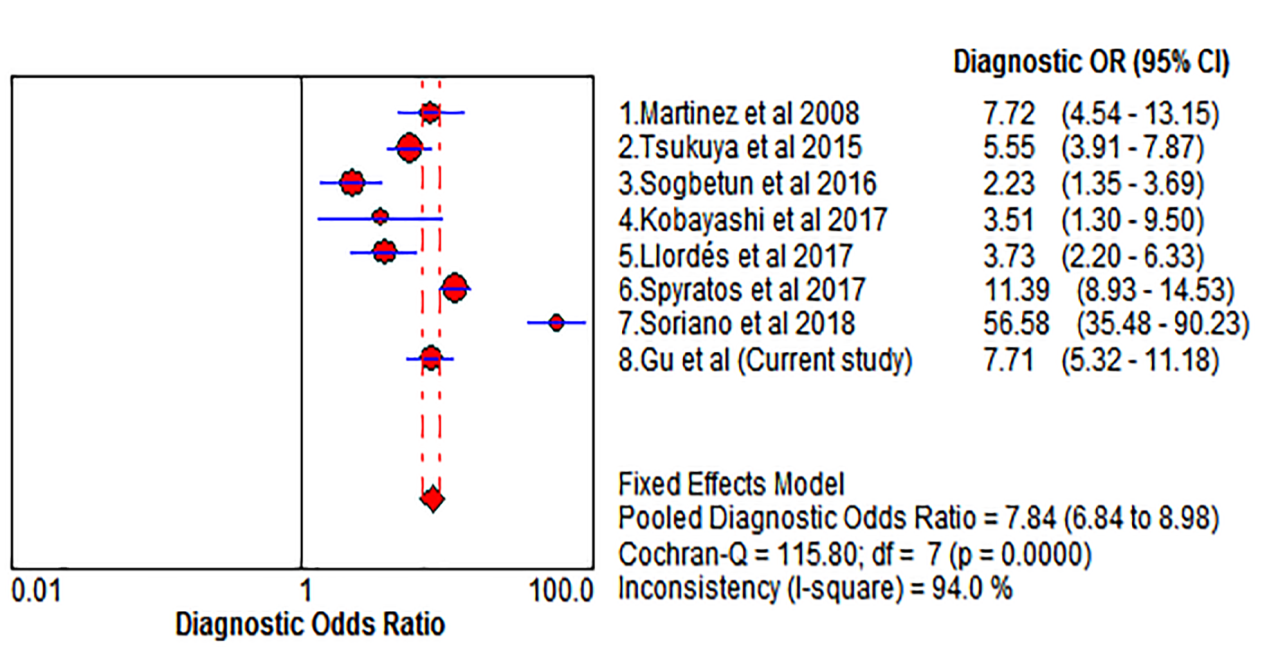


The point estimates of specificity from each study are shown as solid circles. Error bars indicate 95% CIs. I^2^ = (Q-df)/Q.

COPD-PS: Chronic Obstructive Pulmonary Disease Population Screener
